# Investigating the bHLH transcription factor *TSARL1* as marker and regulator of saponin biosynthesis in *Chenopodium quinoa*

**DOI:** 10.1002/jsfa.14436

**Published:** 2025-06-13

**Authors:** Marius Kollmar, Katharina B. Böndel, Lukas John, Stefan Arold, Karl Schmid, David Jarvis, Sandra M Schmöckel, Sophie L Otterbach

**Affiliations:** ^1^ Institute of Crop Science, Physiology of Yield Stability University of Hohenheim Stuttgart Germany; ^2^ Institute of Plant Breeding, Seed Science and Population Genetics University of Hohenheim Stuttgart Germany; ^3^ KAUST Center of Excellence for Smart Health, Biological and Environmental Science and Engineering Division King Abdullah University of Science and Technology (KAUST) Thuwal Kingdom of Saudi Arabia; ^4^ Brigham Young University, Department of Plant and Wildlife Sciences College of Life Sciences Provo UT USA

**Keywords:** *Chenopodium quinoa*, *TSARL1*, mevalonate pathway, breeding marker, qPCR, triterpenoid saponins

## Abstract

**BACKGROUND:**

Quinoa (*Chenopodium quinoa*) is valued for its nutritional benefits and resilience to abiotic stresses. However, its commercial use is limited by bitter‐tasting saponins on the seeds, necessitating resource‐intensive removal processes.

**RESULTS:**

This study demonstrates a single nucleotide polymorphism (SNP), G2078C, in the *Triterpene Saponin Biosynthesis Activating Regulator Like 1* (*TSARL1*) gene, which encodes a basic helix–loop–helix (bHLH) transcription factor, as significantly associated with the non‐bitter phenotype in quinoa. We have developed a PCR assay to demonstrate the pivotal role of this SNP in distinguishing non‐bitter from bitter quinoa varieties, thereby providing a practical tool for breeding and quality control. Our findings confirm the SNP's critical function in downregulating the saponin biosynthesis pathway, through quantitative PCR analyses of *TSARL1*, *TSARL2*, *BAS1*, *CYP716A78* and *CYP716A79*. Furthermore, protein modelling of *TSARL1* validates its responsibility for the bitter phenotype. Investigating early plant development revealed delayed seedling emergence of bitter quinoa and phylogenetic analysis confirmed the bitter allele as the ancestral trait of quinoa.

**CONCLUSION:**

Demonstrating a strong correlation between the non‐bitter phenotype and the G2078C SNP, our study not only validates the SNP's significance but also introduces an efficient method for its detection. This advancement promises to streamline the breeding of non‐bitter quinoa varieties, enhancing the crop's palatability and reducing the need for postharvest processing. Our approach offers significant implications for the agricultural production and nutritional exploitation of quinoa, aligning with efforts to meet global food security and nutritional needs. © 2025 The Author(s). *Journal of the Science of Food and Agriculture* published by John Wiley & Sons Ltd on behalf of Society of Chemical Industry.

## INTRODUCTION

**Figure 1:**
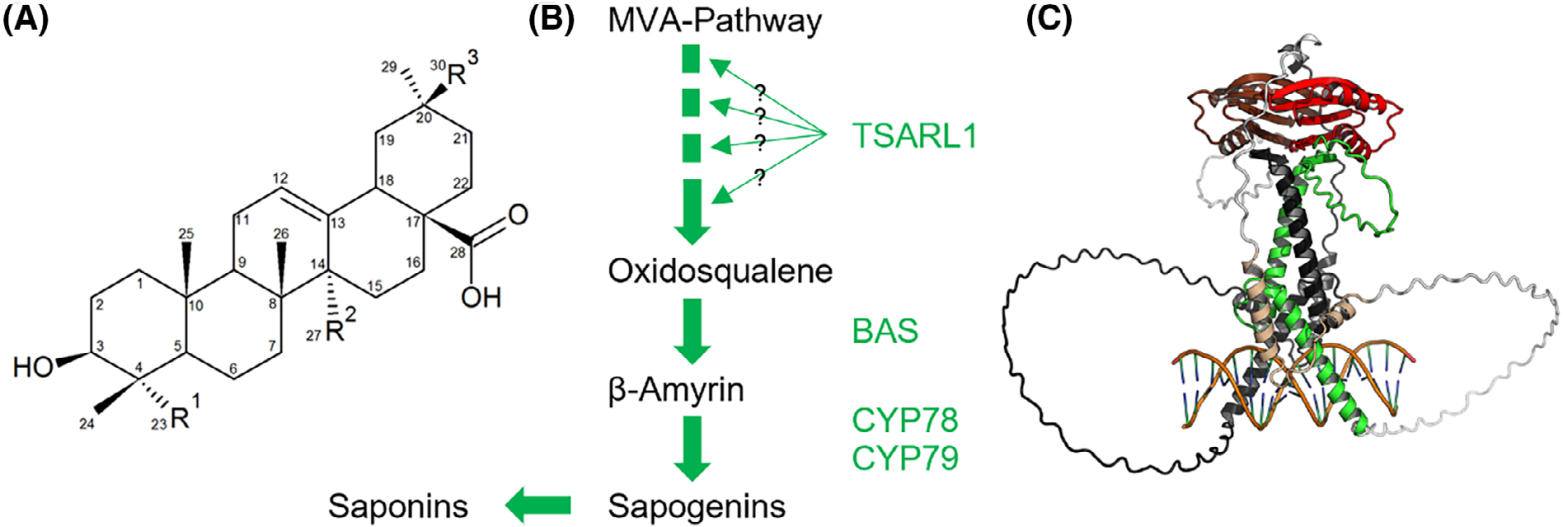
Sapogenin structure, saponin biosynthesis pathway and structural model of TSARL1 in quinoa. (A) Structure of a quinoa sapogenin. Diverse saponins originate from variations in R^1^–R^3^. Sugars are linked at C3 and C28. Modified from Otterbach *et al*.^4^ (B) Schematic representation of the MVA pathway, resulting in saponins. The enzymes TSARL1, BAS, CYP78 and CYP79 are shown in green. (C) AlphaFold3 structural model of a TSARL1 homodimer bound to DNA. One TSARL1 chain is coloured as: green, bHLH domain and the beta strand of the C‐terminal domain that is retained in the non‐bitter isoform. The region lost in the non‐bitter form is shown in red. Flexible regions are coloured white, and structured regions modelled with low certainty are coloured in beige. The second TSARL1 chain is coloured in black, with the region deleted in the non‐bitter accessions in brown. The DNA backbone is coloured in green and the bases in blue.

Quinoa (*Chenopodium quinoa* Willd.) has attracted attention in recent years due to its favourable nutrient composition with essential amino acids, high protein content compared to cereals and tolerance to abiotic stresses, such as salinity and drought.^1^, ^2^ Most quinoa seeds contain bitter‐tasting saponins, triterpenoid glycosides, with antinutritive properties which are removed before human consumption.^3^, ^4^, ^5^ Saponins are complex secondary metabolites comprised of an aglycone backbone (Fig. 1(A)) and sugar moieties. Over 80 different saponins were detected in quinoa,^6^ with aglycone type possibly affecting characteristics like odour and taste. Saponins are predominantly located in the pericarp of quinoa seeds.^7^ Postharvest removal of saponins by extensive washing or mechanical abrasion are labour‐ and water‐intensive and diminish the nutritional value of quinoa seeds.^8^ Consequently, a desirable target is breeding quinoa lines with low/no saponins, which is known as non‐bitter or ‘sweet’ quinoa.

The ability of saponins to form foam in aqueous solutions, due to their hydrophilic glycoside and a lipophilic agylcone, facilitates phenotypic differentiation between bitter and non‐bitter quinoa accessions using the afrosimetric method.^9^ Additionally, gas chromatography allows identification and quantification of saponin aglycones.^4^, ^6^ Quinoa is considered non‐bitter when the pericap saponin content is less than 0.11% of dry seed mass.^10^ Several non‐bitter lines of quinoa have been previously identified, such as 0654 and Atlas.^7^ Jarvis *et al*.^7^ identified a locus associated with the saponin absence on scaffold 3489 chromosome 16 in genome assembly v1 and Cq5B in v2,^11^ which likely regulates saponin biosynthesis in the two analysed bi‐parental populations of bitter quinoa × non‐bitter quinoa.

A gene encoding for a basic helix–loop–helix (bHLH) transcription factor (TF), *TSARL1*, was proposed as a candidate gene regulating saponin biosynthesis.^7^ The bHLH TF family is widespread among eukaryotes, plants, animals and fungi.^12^ They are involved in regulatory networks of plant functions, such as plant growth and development. The family is named after its highly conserved HLH domain, consisting of 60 conserved amino acids with two functionally distinct regions.^13^ The basic N‐terminus contains 13–17 amino acids and is involved in DNA‐binding and specificity. The 45‐amino‐acid‐long C‐terminal HLH region comprises two amphipathic *α*‐helices, required for the formation of homodimeric and/or heterodimeric protein complexes.^14^ Several bHLH TFs were shown to regulate terpene biosynthesis genes: for instance, AtMYC2 from *Arabidopsis thaliana* activates the expression of sesquiterpene synthase^15^ and SlMYC from *Solanum lycopersicum* has been shown to transiently transactivate the terpene synthase promoter in *Nicotiana benthamiana*.^16^ In *Medicago truncatula* MtTSAR1 and MtTSAR2 were found to regulate the transcription of triterpene saponin biosynthesis genes. The overexpression of these genes leads to increased accumulation of triterpene saponins^17^ and they share homology with *TSARL1* in quinoa.

The genes *CqBAS1* (*BAS*), *CqCYP716A78* (*CYP78*) and *CqCYP716A78* (*CYP79*) were found to encode key enzymes in quinoa's saponin biosynthesis pathway.^18^ TSARL1 might exert indirect downregulation on *BAS* and *CYPs* in non‐bitter quinoa accessions (Fig. 1(B)). A hypothesised causal factor for the non‐bitter phenotype in quinoa is the single nucleotide polymorphism (SNP) G2078C located at the end of exon 3 in *TSARL1*.^7^ This SNP appears to alter the regular exon/intron splice boundary, creating a cryptic splice site upstream, leading to alternative splicing and a premature stop codon, and thus likely to a non‐functional truncated protein.^7^

Here, we show that TSARL1 is regulating saponin biosynthesis in quinoa. We developed a PCR assay followed by restriction digest to detect the SNP G2078C, differentiating between bitter and non‐bitter quinoa plants. We observed that in nearly all non‐bitter accessions we can detect the SNP. This simple PCR assay also allows for efficient testing of non‐bitter quinoa plants in a breeding context or quality control for producers and growers. Additionally, we used quantitative PCR (qPCR) to examine the SNP's impact on *TSARL1* expression and of three downstream genes (*BAS* and *CYPs*) involved in saponin biosynthesis. To further understand the role of TSARL1 in the non‐bitter phenotype, we employed protein modelling for functional prediction and conducted a phylogenetic analysis to trace the origin of non‐bitter quinoa accessions and found that bitter quinoa is likely the ancestral state.

## MATERIALS AND METHODS

### Protein analyses and modelling

Gene sequences for scaffold 3489 were retrieved from Jarvis *et al*.^7^ Bitter and non‐bitter protein sequences were aligned using Muscle to locate amino acid differences.^19^ Variants were analysed using the steps outlined previously.^20^ In brief, we used SwissModel^21^ to detect multimeric states, possible ligands and functionally relevant homologues. Three‐dimensional protein structures (including possible multimeric states and ligands) were predicted using AlphaFold3.^22^ Variants were analysed individually using PyMol (pymol.org). Structural homologues were identified with Foldseek.^23^ Transmembrane helices and signal peptides were predicted with Phobius (https://phobius.sbc.su.se/).^24^

### Plant material

The quinoa accessions (Table S1) were received from the United States Department of Agriculture (USDA) and Institute of Plant Genetics and Crop Science (IPK, Gatersleben, Germany). Plants were grown in 12 cm diameter pots with a soil mixture of 3/4 Substrat 5 (Klasmann‐Deilmann GmbH, Geeste, Germany) and 1/4 sand. Plants were grown in a greenhouse (Phytotechnikum, University of Hohenheim, Germany) at temperatures between 25 and 30 °C and 16 h/8 h day/night cycle.

### Foam assay using the afrosimetric method

Saponins have foaming properties in water. This foam height was used as a semi‐quantitative measurement for total saponin content, the afrosimetric method.^9^, ^25^ Five seeds per accession were shaken vigorously with 0.5 mL of distilled water in a 1.5 mL micro‐centrifuge tube for 30 s. The resulting foam height was measured using a calliper.

### Early growth stage development

Early growth stage development of quinoa accessions (Table S2) was evaluated daily in 12 replicates in a randomised complete block design. A seed was considered emerged once its cotyledons became visible and clearly separated. The first true leaves were identified when the initial set of true leaves appeared at an angle of 45° to the stem.

### SNP assay using PCR and restriction enzyme digest

Plant material of two‐week‐old plants was harvested and frozen in liquid nitrogen before DNA extraction. Genomic DNA (gDNA) was extracted from 100 mg of leaf material according to Guo *et al*.^26^

A 50 μL PCR reaction consisted of 1 mmol L^−1^ dNTPs, 1× Taq reaction buffer (Biozyme), 0.25 μmol L^−1^ of each primer (Table S3), 2 mmol L^−1^ MgCl_2_, 1.25 U Taq polymerase (Biozyme) and 2 ng of gDNA. PCR conditions were set to 94 °C for 3 min for initial denaturation, 38 cycles at 94 °C for 15 s, 58.6 °C for 30 s, 72 °C for 1 min, followed by 72 °C for 5 min final extension. An amount of 10 μL of the PCR reaction was visualised by gel electrophoresis (1.5% agarose). An amount of 15 μL of the PCR reaction was incubated with 2 U of HpyCH4IV restriction enzyme (NEB, MA, USA) with 1× CutSmart buffer (NEB, MA, USA) overnight at 37 °C. The DNA fragments were analysed using gel electrophoresis (1.5% agarose).

### Quantitative real‐time PCR

Six quinoa accessions (three bitter and three non‐bitter) were grown with three biological replicates. Samples were taken from leaves, roots and panicles at different developmental stages: bud formation (growth stage BBCH 51–59), anthesis (BBCH 60), seed set 33% and seed set 67% (both BBCH 70–81) (Fig. S1, Table S4).^27^

RNA was extracted from 100 mg of plant tissues using a NucleoSpin RNA Plant and Fungi Minikit (Macherey‐Nagel GmbH & Co. KG, Düren, Germany) according to the manufacturer's instructions, with an additional DNase treatment using a DNA‐free™ DNA Removal Kit (Invitrogen, Carlsbad, CA, USA) at 2 U DNase for 10 μg of RNA. RNA quality was assessed according to MIQE guidelines.^28^ The integrity, concentration and pureness were tested using gel electrophoresis, NanoPhotometer (IMPLEN), Qubit 3.0 fluorometer (Thermo Fisher Scientific, MA, USA; 10% of samples were tested to verify values above 8.0) and PCR.

RNA passing quality assessment was translated into complementary DNA (cDNA) using an iScript cDNA Synthesis Kit (Bio‐Rad, Hercules, CA, USA) according to the manufacturer's instructions. cDNA was tested by PCR and primer efficiency was tested by qPCR (Fig. S2). qPCR was done with a CFX Connect Real‐Time PCR detection system (Bio‐Rad, Hercules, CA, USA) with 8 μL of reaction volume: 4 μL of 2× iTaq Universal SYBR Green Supermix (Bio‐Rad, Hercules, CA, USA), 0.32 μL of forward and reverse primer (200 nmol L^−1^) (Table S3), 1.68 μL of MilliQ‐H_2_O and 2 μL of cDNA (*ca* 100 ng). qPCR conditions were: 95 °C for 3 min for initial denaturation, then 40 cycles at 95 °C for 10 s, 60 °C for 30 s; finally 65–95 °C with 0.5 °C slope for 5 s for melting curve analysis.

### Saponin analysis by gas chromatography–mass spectrometry (GC–MS)

The detection and quantification of saponins was performed indirectly by quantifying the main sapogenin oleanolic acid exactly as described previously.^29^

### Statistical analyses

A linear mixed‐effects model was implemented in the R (Version 4.2.3) package lme4^30^ to dissect the relationships between the saponin phenotype and emergence, formulated succinctly as:

$$Y_{\mathrm{ijk}}=\mu +S_{i}+G_{j}+B_{k}+\left(A_{i}\right)+\epsilon_{\mathrm{ijk}}$$

where *Y*_*ijk*_ denotes the seedling emergence time, *μ* is the intercept, *S*_*i*_ represents the fixed effect of the saponin phenotype, *G*_*j*_ denotes the fixed effect of the site of origin, *B*_*k*_ accounts for the block effect, (*A*_*i*_) signifies the random effect of accession within each saponin phenotype, thus capturing intra‐accession variability, and *ϵ*_*ijk*_ is the residual error.

To further elucidate the influence of the saponin phenotype on emergence time, estimated marginal means (EMMs) were calculated for each level of saponin phenotype using the emmeans package in R.^31^

*Post hoc* pairwise comparisons were conducted to assess the statistical significance of differences between the saponin phenotypes' EMMs. The Sidak method for adjusting *P* values implemented in the R package multcomp^32^ was used to control for the family‐wise error rate in these multiple comparisons.

qPCR data were tested for normal distribution using the Shapiro–Wilk method and for variance homogeneity using Levene's test. Normalised expression levels (NELs) were calculated according to Vandesompele *et al* ^33^ (Table S4). Statistical significance of differences was tested by conducting a *t*‐test to compare NELs between bitter and non‐bitter quinoa accessions across different developmental stages. *Polyadenylate‐Binding Protein 2‐like* (*PAB2*) and *Outer Envelope Pore Protein 16‐3, Chloroplastic/Mitochondrial‐like* (*OEP*) (Table S3) were used as reference genes. Significance levels were visualised using asterisks. The sample size per boxplot ranged from 4 to 9. A threshold for the Cq values was set to 34 cycles. The NEL of samples with a Cq value greater than 34 was set to 0.

Data were visualised using the R package ggplot2.^34^

### Evolutionary analyses

To generate phylogenetic trees of the whole genome and of the *TSARL1* gene, we used publicly available whole‐genome resequencing data of 179 quinoa accessions,^35^ for which we had saponin data. Four *Chenopodium berlandieri* accessions^7^ were added as outgroup to root the trees and infer the ancestral state of the G2078C SNP. In genome assembly version 1, *TSARL1* is mapped on chromosome 16, position 68.549.573 to 68.551.812 (ATG to TAA, + strand), with SNP G2078C at position 68.551.650.^7^ In version 2, *TSARL1 is* located on Cq5B, position 8.944.605 to 8.942.366 (ATG to TAA, − strand), with the SNP G2078C at position 8.942.528.^11^ As *TSARL1* is on the negative strand, here we refer to the reverse complement of the sequence.

Mapping and SNP calling are described elsewhere.^36^ Filtering was done with vcftools v0.1.17^37^ where we set the minimum depth to 5 (−minDP 5), allowed for up to 25% of missing data at a position (−max‐missing 0.75) and removed indels as well as cpDNA, mtDNA and the CqUO scaffold. The *TSARL1* gene was extracted from the whole genome data with vcftools v0.1.17.^37^ Here, we added 5 kb upstream and extracted from the unfiltered VCF to obtain sufficient SNPs for the phylogenetic analysis.

The phylogenetic trees were constructed using the neighbour joining (NJ) method implemented in the R package ape v5.7.1.^38^ First, the VCF was transformed into a DNAbin object with the R package vcfR v1.14.0,^39^ then the allele sharing distance was used to calculate the genetic distance matrix. Finally, we built the NJ tree and conducted 100 bootstrap replications using the functions nj and boot.phylo, respectively. The NJ trees were visualised with FigTree v1.4.4 (http://tree.bio.ed.ac.uk/software/figtree/).

To confirm the clustering in the NJ trees with a non‐phylogenetic method, we conducted principal component analysis (PCA) with the R package SNPRelate v1.34.1.^40^

## RESULTS

### *In silico* support for the dominant role of *TSARL1* for bitter *versus* non‐bitter phenotype

Jarvis *et al*.^7^ performed bulk segregant analyses using two bitter × non‐bitter populations and identified scaffold 3489 with the locus underlying saponin production. They proposed *TSARL1* with a G2078C SNP to be the causative gene – distinguishing bitter from non‐bitter seed. Other genes on scaffold 3489 were not investigated. *TSARL1*'s bitter gene product is a 315‐residue TF. Alternative splicing resulting from the G2078C SNP, that segregates with non‐bitter accessions, leads to the loss of residues 227–315. Structure prediction with AlphaFold3 shows these residues form a folded domain consisting of a beta‐sheet covered by two helices. Moreover, AlphaFold3 predicts this domain forms a homodimer that sits atop of the DNA‐binding dimeric HLH structure (Fig. 1(C)). This domain is specific to plants (Fig. S3). In addition to stabilising the dimeric DNA‐binding conformation of the bHLH TF, its hydrophobic bHLH‐distal site suggests it could recruit additional components. Deletion of this domain would abolish both functions. This makes the G2078C SNP in *TSARL1* likely causative for distinguishing bitter and non‐bitter quinoa. To support this, we investigated other genes on scaffold 3489. We selected those genes that show lower expression in non‐bitter compared to bitter quinoa (based on RNAseq data available from Jarvis *et al*.^7^) and contain at least one SNP.

Contrary to the fundamental difference between bitter and non‐bitter *TSARL1* gene products, substitutions in the other genes are predicted to have no or minor impact on their function. Most noteworthy substitutions are in AUR62017181 (Fig. S4) and AUR62017191 (Fig. S5). AUR62017181 is a horseshoe‐shaped leucine‐rich repeat domain annotated as a disease resistance gene. The D336N (bitter to non‐bitter) is in the concave side, where these types of proteins typically bind their targets. The mutation will not cause steric problems but diminishes the net negative charge at this position, which may influence possible interactors. The second variant in this protein, T381I likely does not cause structural changes, and is situated in an external position where normally ligands do not bind. AUR62017191 has a helical transmembrane domain with a protease‐associated domain forming a lid‐like structure. The T200M substitution is in the transmembrane domain, with an unknown function. However, this relatively conservative mutation should not introduce major changes, but may influence dynamics and fine‐tuning of this domain. Substitutions in the remaining genes are found in solvent‐exposed regions and are likely not linked to functional changes. AUR62017198 (Fig. S6), a possible microtubule motor protein, has a solvent‐exposed E26D variant in the calponin homology domain. In AUR62017200 (Fig. S7), a metallophos domain‐containing protein, the I80M variant is a solvent‐exposed conservative substitution, that does not create clashes. The second substitution, M282K, in the hydrophobic side chain of lysine can mimic hydrophobic contacts of methionine, and NH groups can reach the solvent without clashes. In AUR62017213 (Fig. S8) the G366R substitution is in the demethylase domain. Although G/R is a non‐conservative substitution, it is tolerated as the glycine is in an exposed loop, and the substituting arginine does not cause clashes with its surroundings. Finally, the T52A substitution found in AUR62017228 (Fig. S9), which has homology to glycosyltransferases required for the biosynthesis of heparan sulfate, is in a small domain formed by three beta sheets, following membrane‐anchoring N‐terminal helix. The function of this region is unknown. T42 is part of a hydrophobic surface. Substitution with the hydrophobic alanine is not expected to destabilise this structure, which is also stabilised by an internal disulfide bond. Taken together, these observations strongly support that the variation in *TSARL1* is mostly, if not completely, responsible for the bitter *versus* non‐bitter phenotype.

### SNP G2078C is associated with non‐bitter phenotype

**Figure 2:**
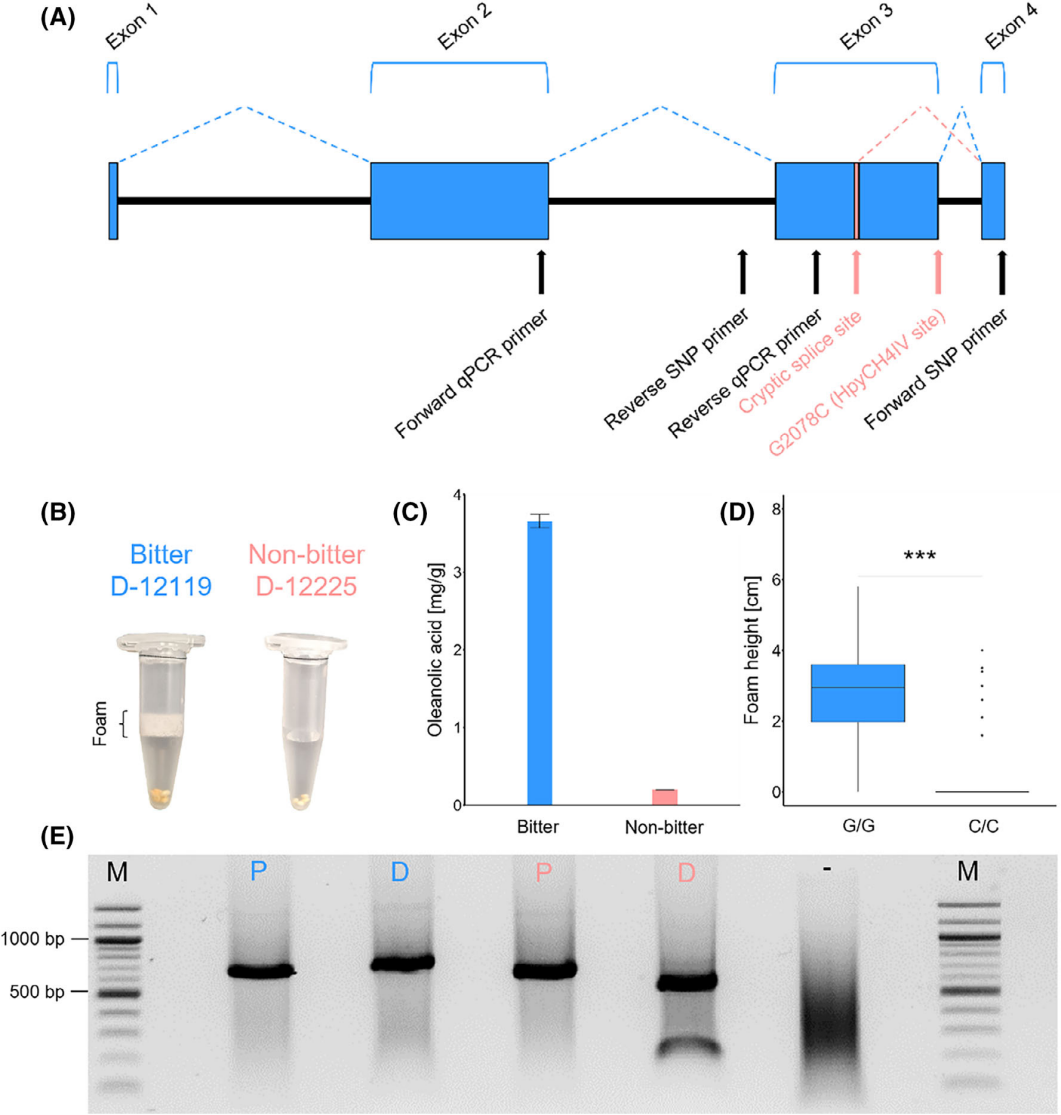
SNP association with non‐bitter quinoa. (A) Schematic representation of *TSARL1*'s gene structure with blue boxes indicating exons and black lines indicating introns. Dashed blue lines illustrate splicing sites in non‐mutated *TSARL1*, while red lines indicate splicing sites when the SNP G2078C is present. This point mutation, indicated by a red arrow, creates a restriction site for the restriction enzyme HpyCH4IV occurring in non‐bitter quinoa accessions. Furthermore, binding sites of the primer pairs used for qPCR and SNP detection are indicated by black arrows. Modified from Jarvis *et al*.^7^ (B) Two quinoa accessions (D‐12119 and D‐12225) showing bitter phenotype with foam (left) and non‐bitter phenotype without foam (right) using the afrosimetric test. (C) Oleanolic acid content in the seed pericarp of the two accessions (*n* = 2) using GC–MS. (D) Foam height of quinoa accessions with the SNP and a G/G allele (*n* = 123) compared to quinoa accessions without the SNP and a C/C allele (*n* = 43). (E) SNP assay: agarose gel electrophoresis of PCR products (680 bp) on the left side (P), and after enzymatic digest on the right side (D). Molecular marker M = 100 bp ladder, samples from bitter quinoa display a single band at 680 bp after digest and samples from non‐bitter quinoa display two bands at 150 and 530 bp.

To test associations between the non‐bitter phenotype in quinoa and the SNP G2078C in *TSARL1* (Fig. 2(A)), we analysed 230 quinoa accessions (Table S1) for the presence of the SNP and their associated saponin content. We used the afrosimetric method, a semi‐quantitative approach where seeds are shaken in water to estimate saponin levels (Fig. 2(B), Fig. S10). These observations correlate with quantified saponin levels as determined by GC–MS (Fig. 2(C)). We developed a SNP assay, based on PCR amplification of a *TSARL1* gDNA fragment, followed by restriction digest detecting the G2078C SNP using the restriction enzyme HpyCH4IV (Fig. 2(A)). Agarose gel electrophoresis of the digested PCR fragment shows two bands for phenotypical non‐bitter quinoa accessions (i.e. no foam) and a single band in phenotypical bitter accessions (i.e. foam) (Fig. 2(D)). Our results show a strong association of non‐bitter phenotype with the occurrence of the SNP in *TSARL1* (Table S1).

We found 156 of 183 accessions align with findings by Jarvis *et al*.^7^: bitter accessions exhibited the G allele and non‐bitter accessions the C allele. Comparing our SNP assay genotyping with sequencing data from Patiranage *et al*.^36^ (Table S1), we found some discrepancies among 183 sequenced accessions. (i) Eight were both genotypically (SNP assay) and phenotypically (foam assay) bitter, yet exhibited the C allele in the sequencing data. Similarly, (ii) seven accessions were classified as non‐bitter both genotypically (SNP assay) and phenotypically (foam assay) but had the G allele in the sequencing data. (iii) Four accessions that identified as bitter by SNP assay (G allele) were phenotypically non‐bitter according to the afrosimetric test. (iv) Eight accessions showed matching SNP assay and foam assay results, while sequencing data identified these as heterozygous (G/C). Particularly, irregularities suggesting a functional *TSARL1* gene in non‐bitter quinoa are interesting because they could indicate other regulatory mechanisms. The occurrence of bitter accessions with a non‐bitter (C/C) SNP genotype might be attributed to segregation since the individual seeds tested via SNP assay and foam assay were not identical to those sequenced.

### Bitter quinoa exhibits delayed seedling emergence

After assessing the correlation of SNP G2078C with the saponin phenotype in quinoa, we investigated its impact on early plant development as this is a critical factor for agricultural productivity.

**Figure 3:**
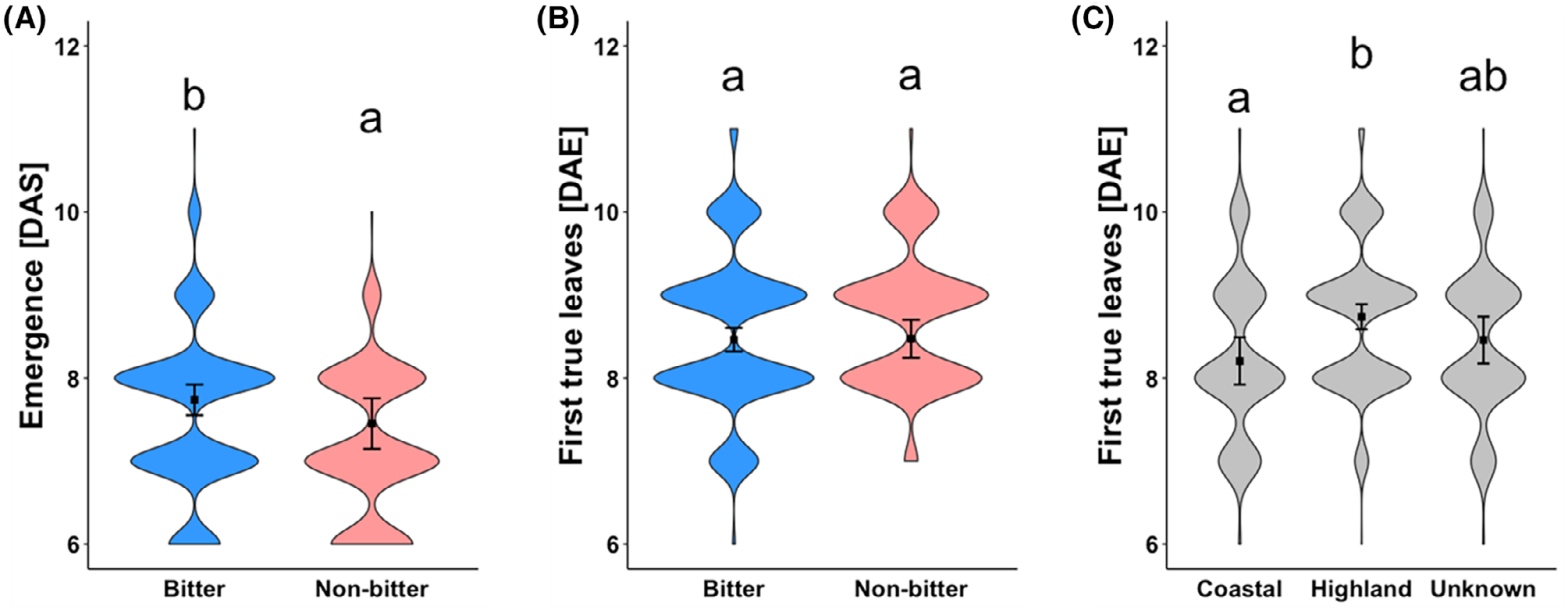
Effect of saponin phenotype and site of origin on early growth stage development. (A) Analysis of days to seedling emergence from sowing (in days after sowing (DAS)), (B) days to first true leaf appearance (in days after emergence (DAE)) depending on saponin abundance and (C) site of origin. *n* = 1799 (175 accessions, 3–12 replicates each) (Table S2). Vertical error bars denote upper and lower confidence limits adjusted using Sidak's method for multiple comparison corrections. Data points are jittered for clarity and individual observations are shown as dots, providing a visual representation of data point distribution across group levels. Groups sharing the same letter are not significantly different (*P* = 0.05).

Bitter quinoa exhibited a slower emergence rate compared to non‐bitter quinoa (Fig. 3(A)). This finding suggests that saponin presence, associated with the bitter phenotype, may have a delaying effect on germination because saponins could pose a physical barrier during germination.

Contrastingly, early vegetative growth from seedling emergence to appearance of first true leaves showed no significant difference between bitter and non‐bitter accessions (Fig. 3(B)). However, significant differences were observed in early vegetative growth between accessions originating from a coastal site and those from a highland site (Fig. 3(C)). This suggests that the site of origin is a more critical factor for leaf development than saponin presence.

### *TSARL1* SNP is linked to saponin gene regulation

To investigate expression patterns of key enzymes in the saponin biosynthesis pathway, we performed qPCR analysis. Effects of the SNP on expression of *TSARL1*, *TSARL2* and key enzymes in saponin biosynthesis, namely *BAS1*, *CYP716A78* and *CYP716A79*, across three different tissues (floral, leaf and root) and four developmental stages (bud formation, anthesis, seed set at 33% and seed set at 67%) (Fig. S1), as well as embryo samples were determined.

**Figure 4:**
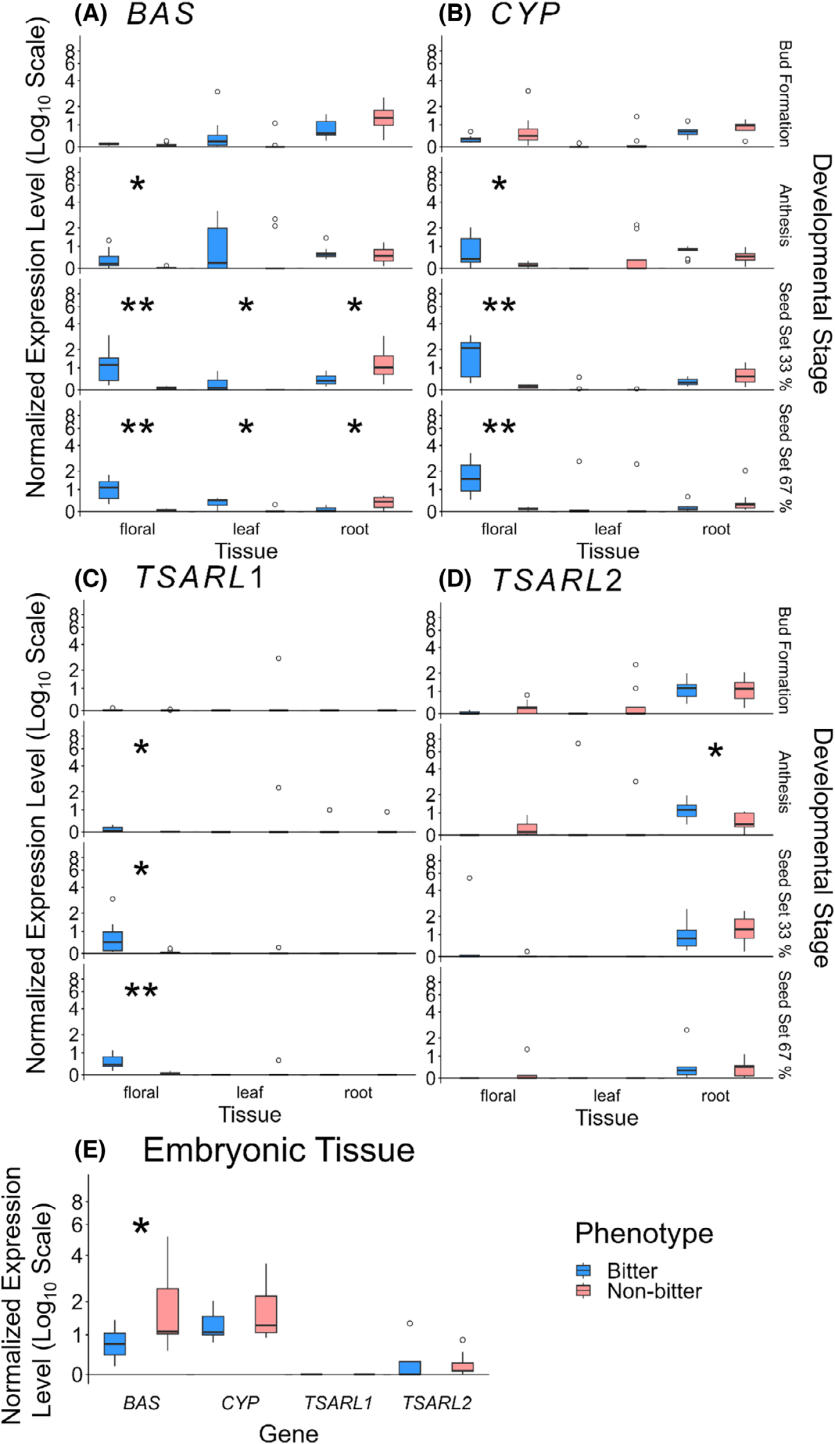
Distribution of gene expression levels in bitter and non‐bitter quinoa accessions for genes involved in saponin biosynthesis. NELs are shown for bitter quinoa accessions (blue) and non‐bitter quinoa accessions (red). Three tissues (floral, leaf and root) and four developmental stages (bud formation, anthesis, seed set 33% and seed set 67%) were analysed. Gene expression levels of (A) *BAS1*; (B) averaged NELs of *CYP716A78* and *CYP716A79* (Fig. S11(B),(C) shows expression levels for each individual CYP); (C) *TSARL1*; and (D) *TSARL2*. (E) Expression levels of aforementioned genes in the embryonic tissue. For each boxplot *n* = 4–9. Significance levels, tested by *t*‐test, are indicated by *P ≤ 0.05 and **P ≤ 0.01.

Gene expression analysis revealed distinct patterns for genes encoding saponin biosynthesis enzymes across different tissues and developmental stages. Our analysis revealed significant expression differences in three key saponin biosynthesis genes in the floral tissue, particularly during seed set stages. *β‐Amyrin Synthase* (*BAS1*), showed no significant expression differences between bitter and non‐bitter quinoa accessions during bud formation (Fig. 4(A)). However, during anthesis and seed set stages, bitter quinoa displayed significantly higher *BAS1* expression, particularly in the floral tissue, a trend similarly observed in leaves. Conversely, root tissues exhibited higher *BAS1* expression in non‐bitter quinoa during seed set. *Cytochrome P450 Oxidases* expression was significantly higher in the floral tissues of bitter quinoa accessions during anthesis and seed set (Fig. 4(B); individual expression of *CYP716A78* and *CYP716A79* displayed in Fig. S11). *TSARL1* expression was barely detectable in non‐bitter quinoa across all tissues and growth stages (Fig. 4(C)). *TSARL1* expression was significantly higher in the floral tissue of bitter quinoa during anthesis and the seed set stages. It should be noted that qPCR primers were designed such that they do not distinguish between bitter and non‐bitter accessions; however, the trend remains the same when primers are designed to only bind to mutated *TSARL1* (Fig. S11). Through the G to C conversion a cryptic splice site is introduced^7^ resulting in a premature stop codon in the mRNA. Consequently, nonsense‐mediated decay^41^ may result in degradation of *TSARL1* mRNA in non‐bitter quinoa, thus leading to undetectable expression levels in qPCR assays. This likely results in an indirect downregulation of the mentioned saponin biosynthesis genes, as the absence of TSARL1 results in a downregulation of the MVA pathway and a downstream expression decrease of *BAS1*, *CYP716A78* and *CYP716A79*.

*TSARL2* expression, predominantly observed in root tissues, was significantly higher in bitter quinoa during anthesis (Fig. 4(D)). The trends described here are also represented in the amount of oleanolic acid measured in two bitter and non‐bitter accessions (Fig. S12). Analysis was performed using GC–MS, which involved a hydrolysis and, thus, quantifications were based on the sapogenin oleanolic acid. Analysis of gene expression in embryonic tissue (of mature seeds) identified a higher expression of *BAS1* in non‐bitter quinoa embryos compared to bitter ones (Fig. 4(E)). No significant differences in expression were observed for other genes in embryonic tissues.

Overall, our findings demonstrate that genes involved in saponin biosynthesis are expressed at higher levels in the floral tissue of bitter quinoa accessions, starting from anthesis and continuing through the seed set stages.

### Bitter allele confirmed as quinoa's ancestral trait

**Figure 5:**
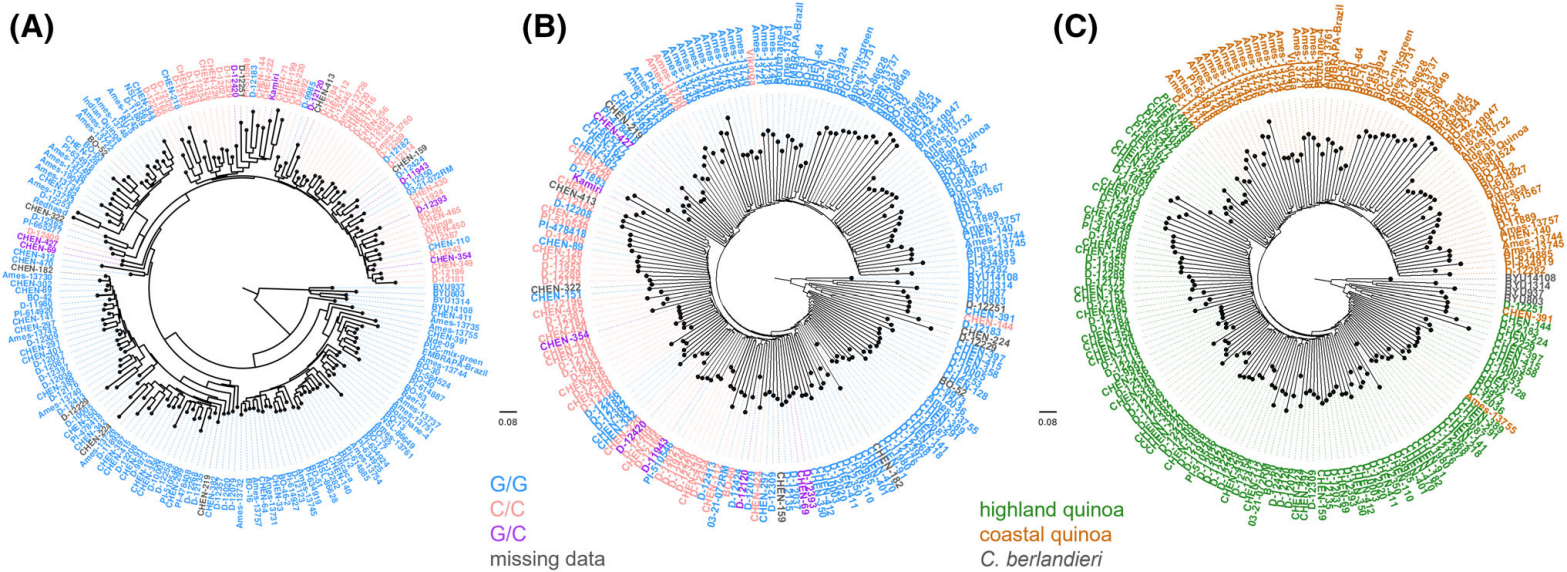
NJ tree. (A) NJ tree constructed from 133 SNPs in the *TSARL1* gene region on chromosome Cq5B (bp 8.942.366 to 8.949.605) and rooted with four *C. berlandieri* accessions. Accessions are coloured according by genotype at position 8.942.528: G/G (bitter) in blue, C/C (non‐bitter) in red and G/C in purple. Accessions with missing data at position 8.942.528 in grey. (B) NJ tree constructed for the whole genome (1 867 553 SNPs) and rooted with the four *C. berlandieri* accessions. Accessions are coloured as in (A) by genotype at position 8.942.528. (C) NJ tree constructed for the whole genome (1 867 553 SNPs) and rooted with the four *C. berlandieri* accessions. Accessions are coloured according to genetic group as identified in the PCA: highland in green, coastal in orange and the four *C. berlandier*i samples in grey.

To explore the evolutionary history of the SNP G2078C in quinoa, we constructed phylogenetic trees to ascertain whether the C allele associated with the non‐bitter quinoa phenotype arose once or multiple times independently. In the tree of the TSARL1 gene, we observed that accessions homozygous for the C allele do not form a monophyletic clade (Fig. 5(A)). For instance, while certain accessions such as D‐12409 (C/C) and CHEN‐427 (G/C) fall outside of this clade, others like D‐12183 (G/G) and CHEN‐110 (G/G) are found within it. This suggests a complex phylogenetic relationship of the TSARL1 gene among non‐bitter and bitter quinoa accessions. Bootstrap support for almost all nodes is very low which is not unexpected due to a low number of SNPs (133): only 6% (11 out of 183) have a bootstrap support of 50 or higher and only 1.6% (3 out of 183) have a bootstrap support of 80 or higher.

A similar pattern is observed for the whole genome tree (Fig. 5(B),(C)) although there are more G/G accessions found within the C/C accession clade and more C/C or G/C accessions (CHEN‐144, Vikinga, Ames‐13760, Ames‐13728 and CHEN‐427) are found outside of it. Most nodes are well supported with 90% (165 out of 183) having a bootstrap support of 50 or higher and 67% (123 out of 183) having a bootstrap support of 80 or higher. This difference to the gene tree is not unexpected, because genealogical processes at individual genes may differ from genome‐wide patterns, e.g. because of selection.

A PCA of the *TSARL1* gene region confirms clustering of accessions which are homozygous for the non‐bitter genotype (C/C), with a mix of G/G and G/C genotypes either within the cluster or in proximity (Fig. S13(A)). The whole‐genome PCA shows two genetic clusters which based on geographic origin information can be classified as highland and coastal (Fig. S13(D)). This agrees with earlier findings.^35^ Here, accessions with the non‐bitter genotype (C/C) are primarily found in the lower half of the highland accession cluster (EV2 < 0) with some accessions in the upper half and only three accessions clustering with the coastal accessions (Fig. S13(C)).

Furthermore, a closer inspection of SNP and genotype frequencies at G2078C provides insight into the ancestral state in quinoa. Among 179 quinoa accessions, 170 have data for this position, and all four *C. berlandieri* accessions are homozygous for the G allele, indicating that G is the ancestral state. The distribution of alleles among quinoa accessions shows a predominance of the G allele (123 accessions homozygous for G, 72% of the total) over the C allele (43 accessions, 25%) and eight heterozygous accessions (4.7%). This corresponds to allele frequencies of 0.741 (246 alleles) for the G allele and 0.259 (86 alleles) for the C allele.

## DISCUSSION

We established *TSARL1*'s role in saponin biosynthesis in quinoa and developed a SNP assay, detecting G2078C as a robust molecular marker for the non‐bitter phenotype. We found differential expression patterns of key genes involved in saponin biosynthesis, validating *TSARL1*'s critical regulatory function.

Our study establishes a clear connection between SNP G2078C in the gene encoding for *TSARL1* and the non‐bitter phenotype in quinoa, by analysing the SNP in 230 quinoa accessions. Our results highlight the potential of utilising this SNP as an effective molecular marker in breeding programmes focusing on cultivating non‐bitter quinoa varieties. This key role of *TSARL1* is supported by Trinh *et al*.^42^ who used Ethylmethansulfonat mutation in the accession Titicaca to knock out *TSARL1* expression resulting in the absence of saponins in seeds.

Interestingly, we observed discrepancies in seven accessions, where the SNP assay indicated a bitter genotype contrary to the non‐bitter phenotype suggested by the afrosimetric shake test. This discrepancy highlights limitations of the afrosimetric method as a semi‐quantitative tool for estimating saponin levels. It is possible that these accessions may produce saponins, but either at levels too low to produce foam or the types of saponins present in these accessions do not form foam. It may also be that the absence of saponins is controlled by another gene. Our findings indicate that the afrosimetric method reliably detects the bitter phenotype when the oleanolic acid content exceeds 0.9 mg g^−1^ (Table S1). It might also be possible that other factors/genes are regulating saponin biosynthesis that we did not detect here. It is known from apple that the TF MYB66 plays a role in the activation of biologically active lupane‐type triterpenes.^43^ In *Withania somnifera*, using a reverse genetics virus‐induced gene silencing approach, the TF *WsWRKY1* was detected to specifically regulate the sterol part of the triterpenoid pathway.^44^ Furthermore, a set of bHLH‐type TFs were shown to promote triterpene biosynthesis in specific cells of *Arabidopsis thaliana*.^45^ Likewise, the bHLH TFs TSAR1 and TSAR2 were identified to regulate triterpene saponin biosynthesis in *Medicago truncatula*.^17^ Finally, the clade IVa bHLH TF TSAR3 was described to regulate triterpene saponin biosynthesis specifically in *Medicago* seeds.^46^

Here, we identified the bHLH TF TSARL1 to regulate triterpene saponin biosynthesis in the floral tissue of *C. quinoa*. Although it is unknown when saponin biosynthesis in quinoa reaches its maximum, saponins can be detected already a few days after anthesis.^7^ Gene expression patterns of key saponin biosynthesis genes *BAS1*, *CYP78* and *CYP79* suggest a peak in saponin biosynthesis during early seed set. Furthermore, it is unknown how saponins get onto the seeds, i.e. in which cells saponin biosynthesis in quinoa takes place. No expression of the respective genes is detected in leaves. Thus, it is unlikely that saponins are produced and transported from leaves to seeds. Since saponins are likely located in the outer seed layer, which is maternal in origin, biosynthesis likely occurs there. It is less probable that saponins could occur in the embryo due to the lack of higher *BAS* and *CYP* expression in embryos of bitter quinoa accessions, and *TSARL1* not being expressed at all in the embryo.

TSARL2 is a potential regulator of saponin biosynthesis in quinoa roots, similar to TSAR3 in *Medicago* seeds,^46^ as indicated by the expression of key genes such as *BAS1*, *CYP78* and *CYP79*. Notably, there were few significant differences in the expression of saponin biosynthesis‐related genes between bitter and non‐bitter accessions in roots, suggesting saponins might be expressed in roots also, even in non‐bitter quinoa (Fig. S11).

Our results suggest that bitter quinoa has a delayed emergence compared to non‐bitter quinoa, though no differences were observed in early leaf development post‐emergence. Previous studies report varying effects of saponins from different plant species on seed germination. Some suggest saponins facilitate germination under stress conditions,^47^ while others report inhibitory effects^48^ or no effect, depending on the species treated.^49^ However, there is a notable lack of research investigating the influence of quinoa saponins on germination and related traits, such as cotyledon emergence. It is possible that saponins provide a physical barrier that delays germination or influences other hormonal factors.^50^ This potential physical barrier could explain delayed emergence observed in bitter quinoa, supporting the need for further research on how saponins impact early vigour in quinoa.

To explore the evolutionary background of the G2078C SNP and the non‐bitter genotype we performed phylogenetic inference and included four *C. berlandieri* samples. *C. berlandieri* is a close relative and ancestor of quinoa.^7^ All four *C. berlandieri* samples are of the G/G genotype indicating that the G allele is ancestral. However, it may be possible that this SNP also segregates within *C. berlandieri*, and by chance, none of the four accessions included here carries a C allele. A PCA revealed that most non‐bitter C/C accessions are in the highland group indicating that the SNP arose in the Andean highlands. The clustering of the C allele in the highland group in both gene‐specific and genome‐wide analyses strongly suggests that the C allele emerged only once.

Small‐seeded varieties with low saponin content were introduced into the Lake Titicaca region of Bolivia and Peru as part of a breeding programme.^51^ However, the programme was discontinued in the highlands due to low acceptance by farmers, primarily because of increased vulnerability to bird attacks.^52^ This historical context suggests that while there may have been some selection for lower saponin content, it was not sustained over time, potentially due to the trade‐off between reduced labour for saponin removal and increased risk of crop loss. This scenario might explain why the SNP has not become more prevalent and now the SNP remains part of the standing genetic variation within quinoa.^53^

In conclusion, our analyses suggest that the C allele at TSARL1 is ancestral. Given the incomplete association of the SNP with saponin content and the observed geographic distribution of bitter and non‐bitter landraces drift, selection by farmers and agricultural practices (e.g. seed exchange, outcrossing between bitter and non‐bitter landraces) played a role in evolutionary history of the TSARL1 gene. The impact of domestication and breeding on this trait's prevalence and expression remains a subject for further investigation. The non‐bitter quinoa accessions show no apparent phenotypic disadvantages compared to bitter accessions, i.e. both types of plants look similar. Essential metabolic pathways appear to function properly in non‐bitter quinoa. Although the MVA pathway is downregulated in the floral tissue in non‐bitter seeds^7^ phytosterols can be measured.^54^ Possibly, metabolites essential for these pathways are produced in and transported from other tissues (such as leaves) of non‐bitter quinoa to the floral tissue. This could compensate for the downregulation of the MVA pathway in the floral tissue. Of note, we did not identify saponins in the leaves using GC–MS (Fig. S12). While several studies report saponins in leaves,^18^, ^55^ this could be due to phytosterols, which are also detected using the vanillin–sulfuric acid assay. Le *et al*.^56^ highlighted limitations in the vanillin–sulfuric acid assay, a common colorimetric method for saponin detection. They noted that this assay is not entirely specific to saponins and can react with other steroidal compounds, including phytosterols.

Taken together, our study presents evidence that the SNP can be used as a marker for saponin presence in the seed pericarp of quinoa. *TSARL1* seems to be downregulated in the floral tissue of non‐bitter quinoa accessions. This interplay of genetic and metabolic factors underlines the complexity of saponin biosynthesis and its regulation, opening paths for further research and their implications for quinoa breeding.

## AUTHOR CONTRIBUTIONS

SLO, MK, SMS designed the study. SLO, MK, LJ, SMS performed the experiments and acquired the experimental data. SLO, MK, LJ, KBB, STA analysed the data and contributed figures. All authors contributed to the interpretation of the data. SLO, MK and SMS drafted the manuscript. All authors reviewed and approved the final manuscript.

## FUNDING INFORMATION

Research was funded by the Deutsche Forschungsgemeinschaft (DFG, German Research Foundation) 495517445 and by the Bundesministerium für Bildung und Forschung (BMBF; research grant code: 02WPM1655), which is part of the PRIMA initiative Quinoa4Med. The research by STA was supported by the King Abdullah University of Science and Technology (KAUST) through the baseline fund, and the KAUST Center of Excellence for Smart Health (KCSH), under award number 5932.

## TEXT EDITING

ChatGPT (Default GPT‐4) was used to correct the text for grammar and edit the text to shorten it without changing content. The authors retain full responsibility for the content.

## Supporting information


**Data S1.** Supplementary Figures.

**Table S1.** *C. quinoa* accessions with information on origin, supplier, sequencing information, phenotype, genotype, foam data and GC–MS values. ‘b’ and ‘s’ indicate bitter and non‐bitter (sweet) quinoa accessions respectively.

**Table S2.** Scoring of emergence time and true leaf development. Table contains data on investigated accessions, categorised by saponin phenotype and site of origin, along with recorded scores for emergence time and the appearance of first true leaves.

**Table S3.** Sequences for genes with primer pairs used for qPCR. ‘s/b’ and ‘b’ indicate that primer pairs were used to either bind to both bitter and non‐bitter quinoa *TSARL1*, or only the non‐mutated *TSARL1* present in bitter quinoa accessions.

**Table S4.** qPCR samples and quinoa accession information. Table shows information about the samples used for qPCR regarding gene, tissue, phenotype, developmental stage, replicate, Cq‐value, Q (relative quantity of gene expression), NF (normalization factor), and NEL (normalized expression level). Furthermore, quinoa accessions of which samples were taken are described regarding accession name, phenotype, origin, accession number, and supplier.

## Data Availability

All data are available within the figures or supplementary material.
