# Supplementary material for: Investigating the bHLH transcription factor TSARL1 as marker and regulator of saponin biosynthesis in Chenopodium quinoa
Source: J Sci Food Agric. 2025 Jun 13;105(13):7329–39. doi: 10.1002/jsfa.14436 (PMC12439090; doi:10.1002/jsfa.14436)
Supplement: Supplementary file 1 — Data S1. Supplementary Figures. [file JSFA-105-7329-s004.docx]

**Supporting Information**


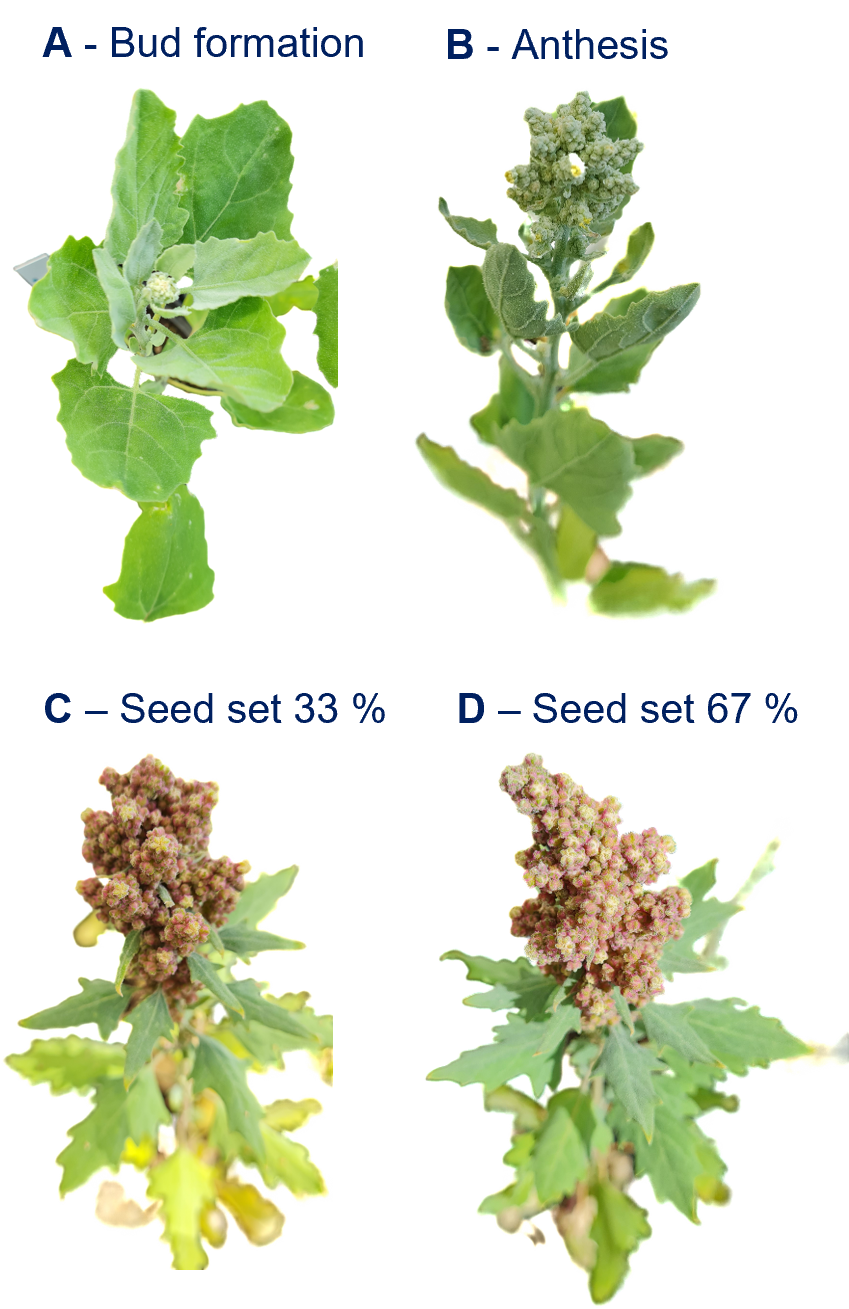


**Figure S1: Developmental stages in which tissue samples for qPCR were harvested**.

Plants were grown to four different developmental stages: **A**, bud formation; **B**, anthesis; **C**, seed set 33 %; **D**, seed set 67 % and samples were taken from three different tissues (floral tissue, leaves, and roots).


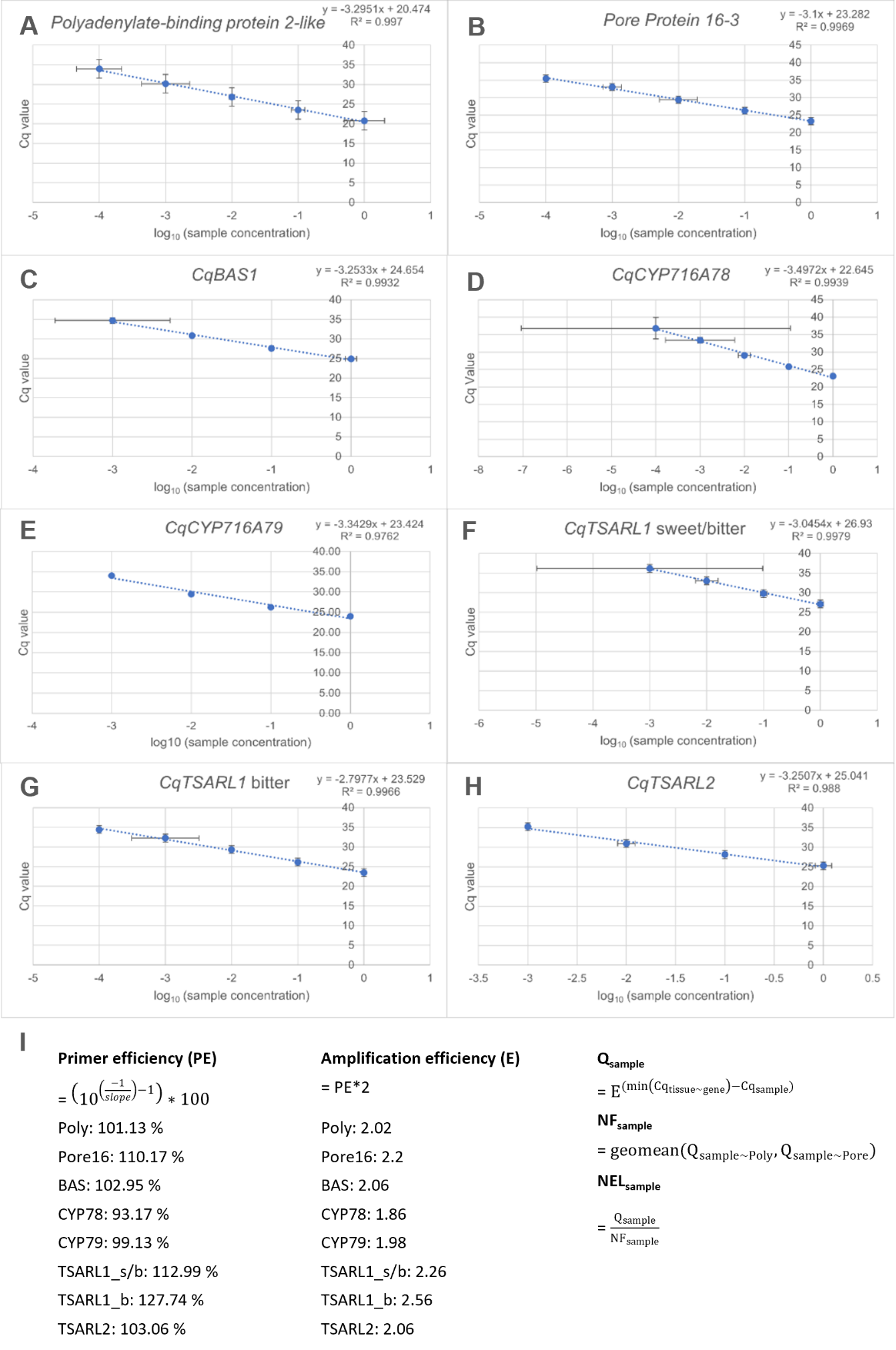


**Figure S2: Standard curves for qPCR primer efficiency of selected genes and calculations of primer efficiency and normalised expression levels.** The standard curve of the reference genes (**A** and **B**) and the genes of interest (**C**-**H**, ‘non-bitter/bitter’ and ‘bitter’ indicate that primer pairs were used to either bind to both bitter and non-bitter quinoa *TSARL1*, or only the non-mutated *TSARL1* present in bitter quinoa accessions) used for qPCR experiments are shown. Furthermore, formulae for calculating primer efficiency (PE), amplification efficiency (E), relative quantity of gene expression (Q), normalisation factor (NF), and normalised expression levels (NEL) are shown. Q is calculated for each sample by subtracting the Cq-value of a specific sample by the minimum Cq-value of all samples of the respective gene and tissue. NF for a specific sample is calculated by taking the geometric mean of the respective Q-values of both reference genes. Finally, NEL for a specific sample is calculated by dividing that samples Q-value by its NF value.


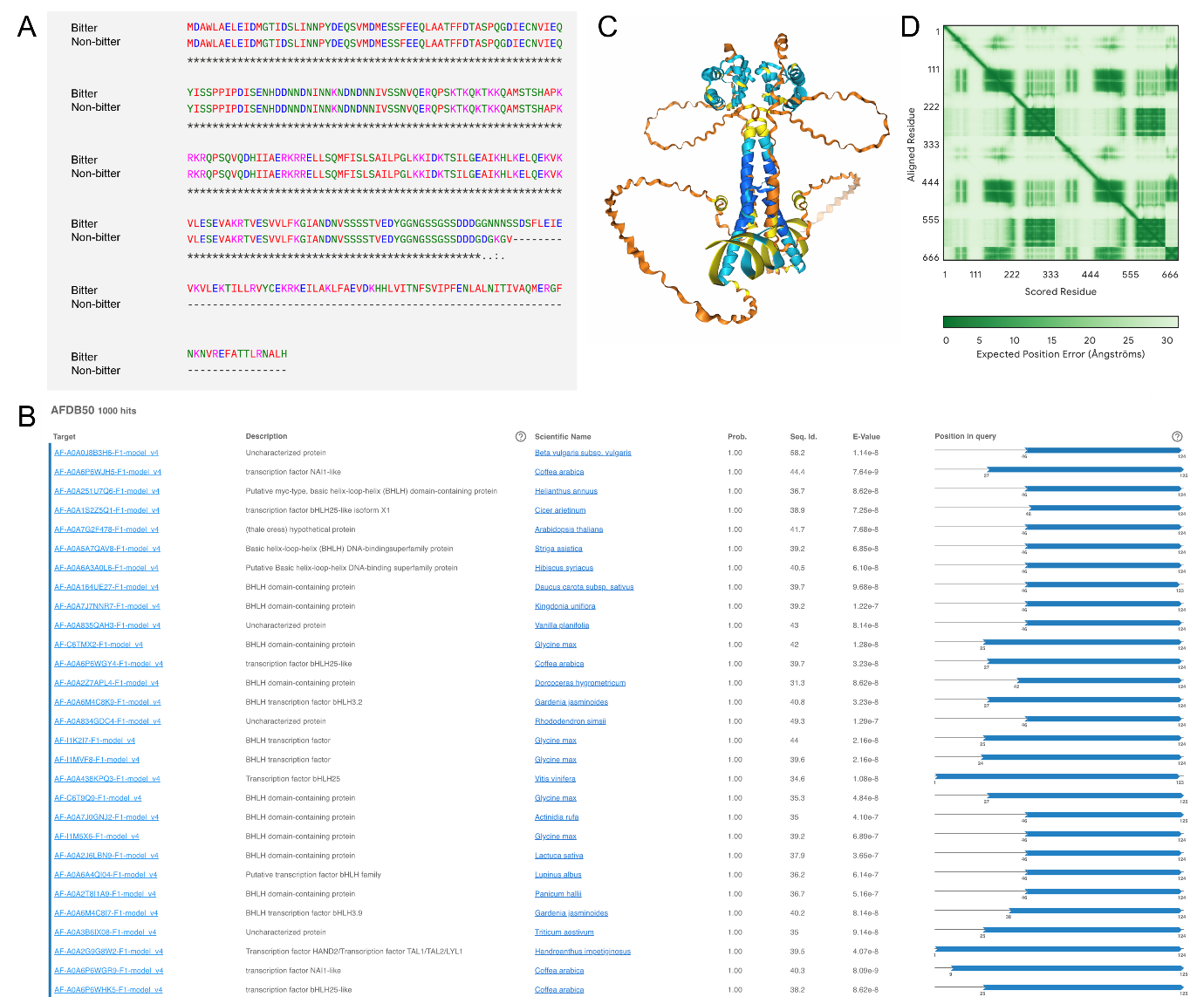


**Figure S3: AUR62017204 (TSARL1).** **A**, Sequence alignment. **B**, Foldseek result for the C-terminal domain. **C**, pLDDT-colored AlphaFold model of Figure 2. **D**, Corresponding predicted aligned error (PAE) plot (first both protein chains, then both DNA sequences, GAGTAGCACGTGCTACTC), supporting the dimeric model and DNA interactions.


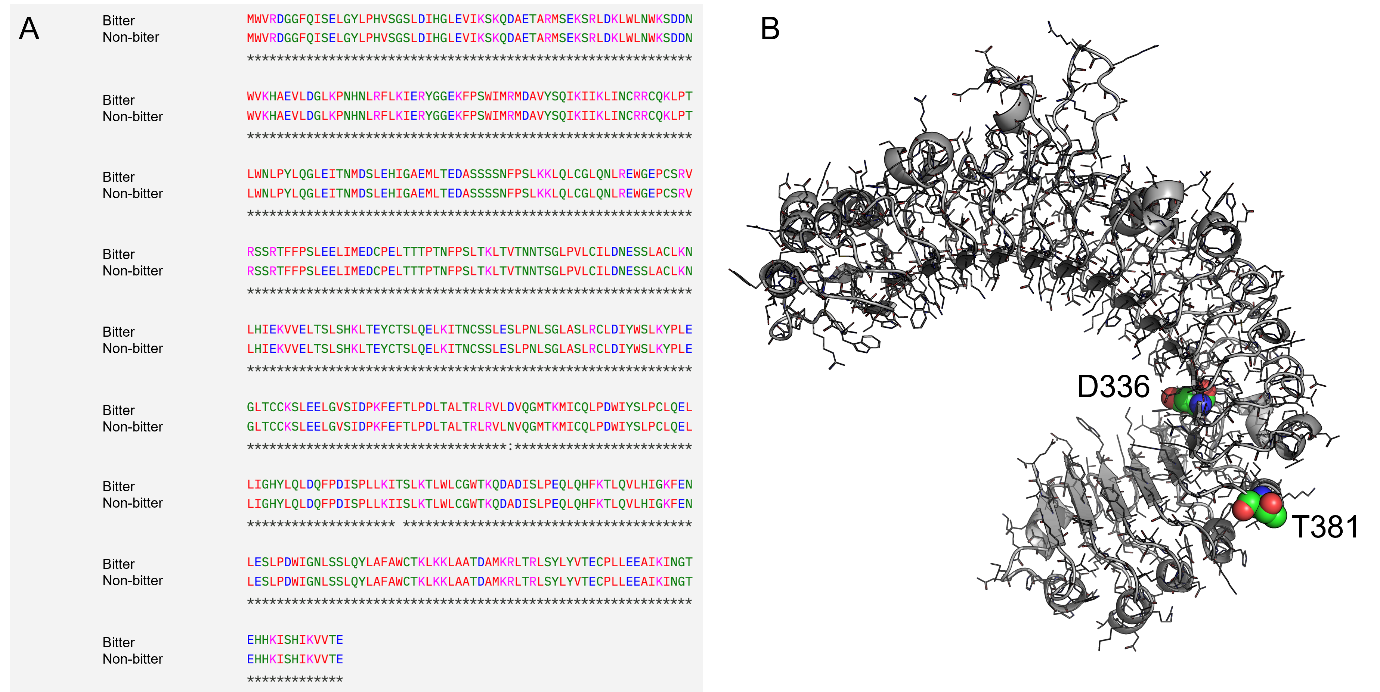


**Figure S4: AUR62017181.** **A**, Sequence alignment. **B**, AlphaFold model of the bitter form. Substituted residues are shown as coloured sphere models.


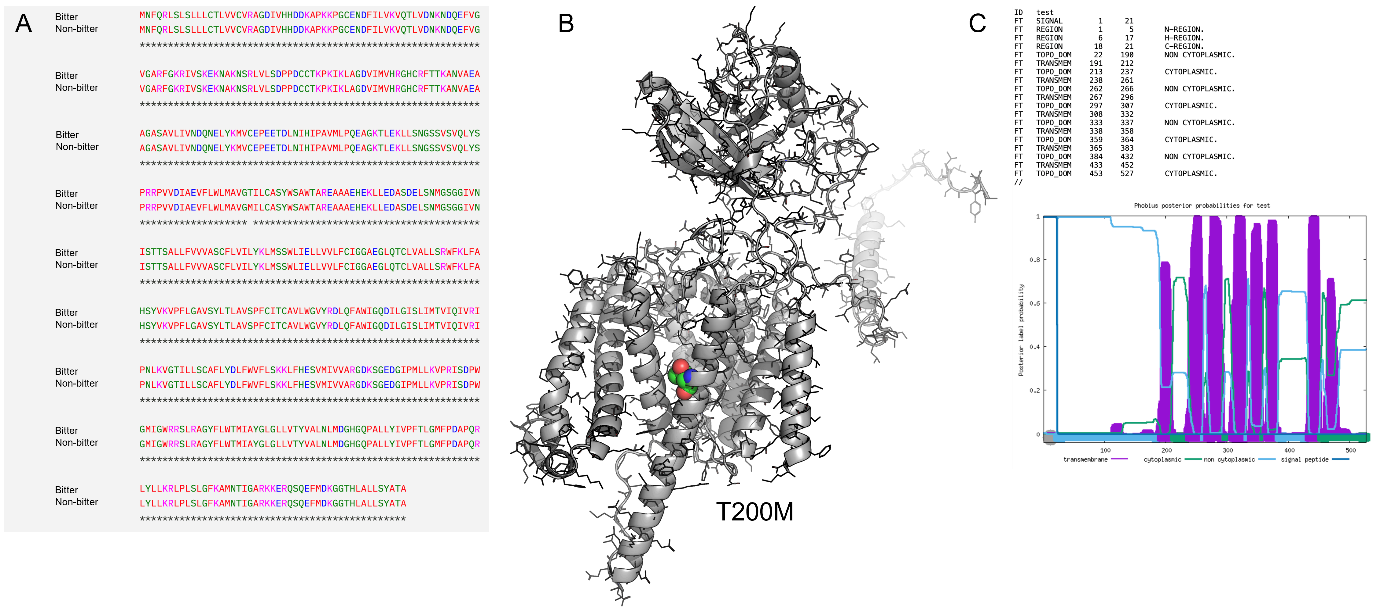


**Figure S5: AUR62017191.** **A**, Sequence alignment. **B**, AlphaFold model of the bitter form. Substituted residues are shown as coloured sphere models. **C**, Trans-membrane helix prediction by Phobius.


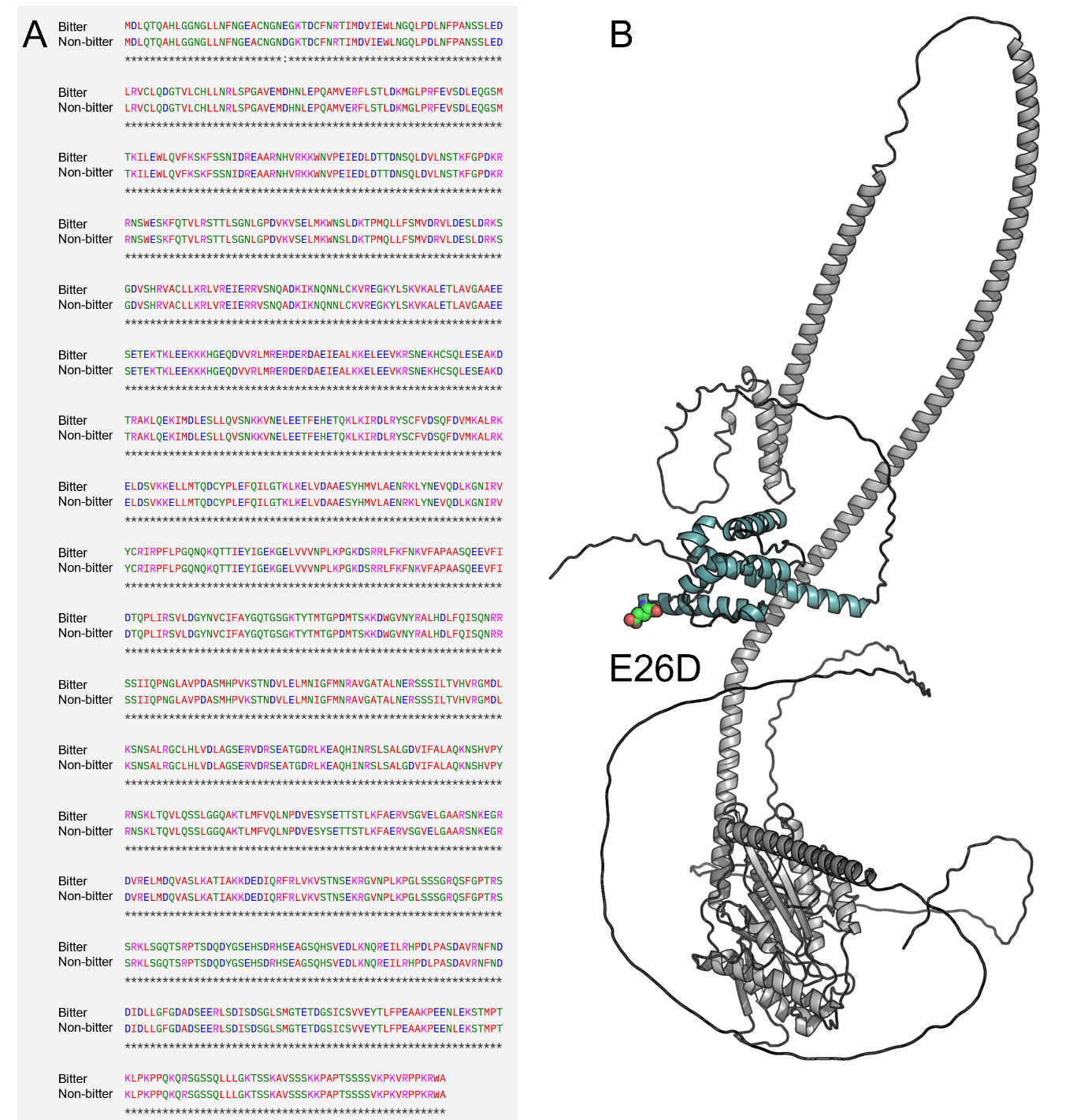


**Figure S6: AUR62017198.** **A**, Sequence alignment. **B**, AlphaFold model of the bitter form. Substituted residues are shown as coloured sphere models.


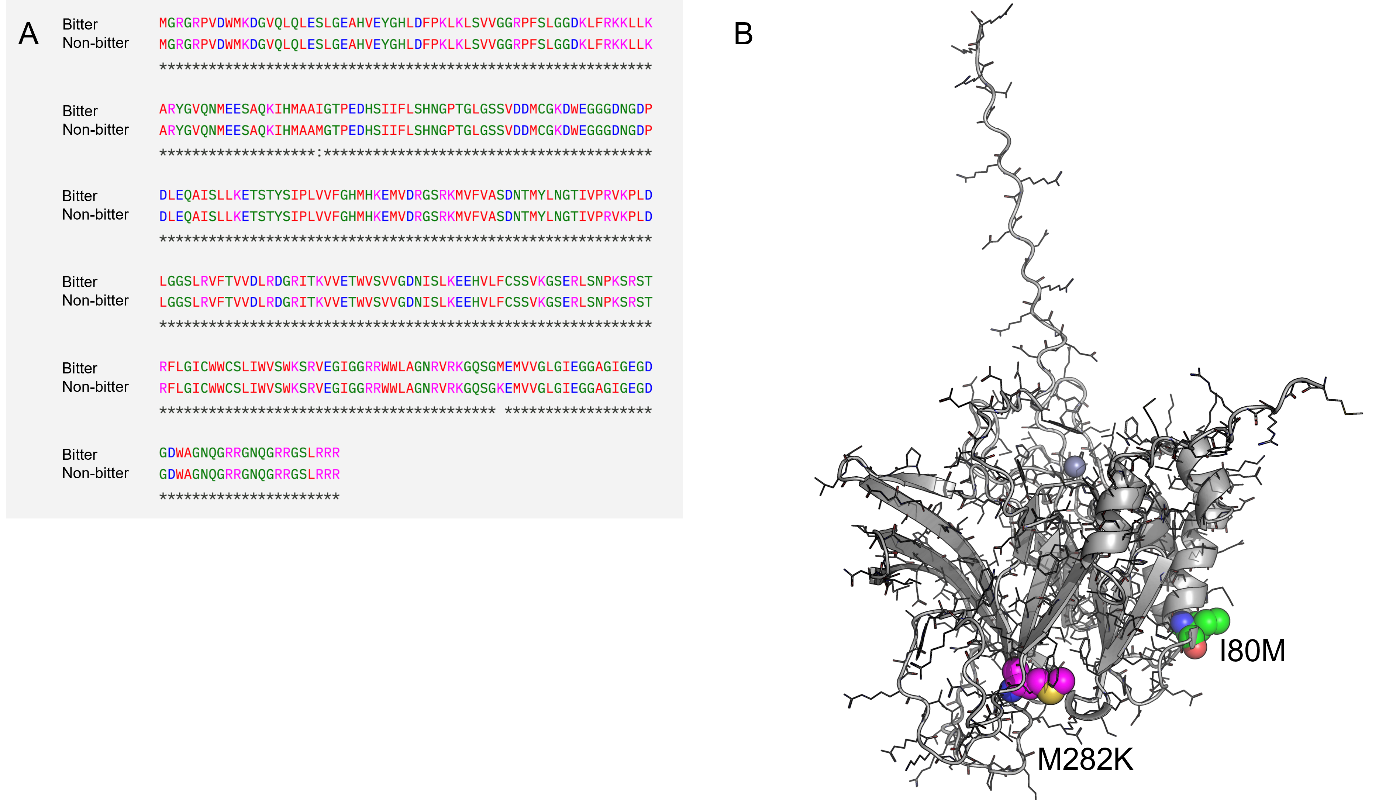


**Figure S7: AUR62017200.** **A**, Sequence alignment. **B**, AlphaFold model of the bitter form. Substituted residues are shown as coloured sphere models.


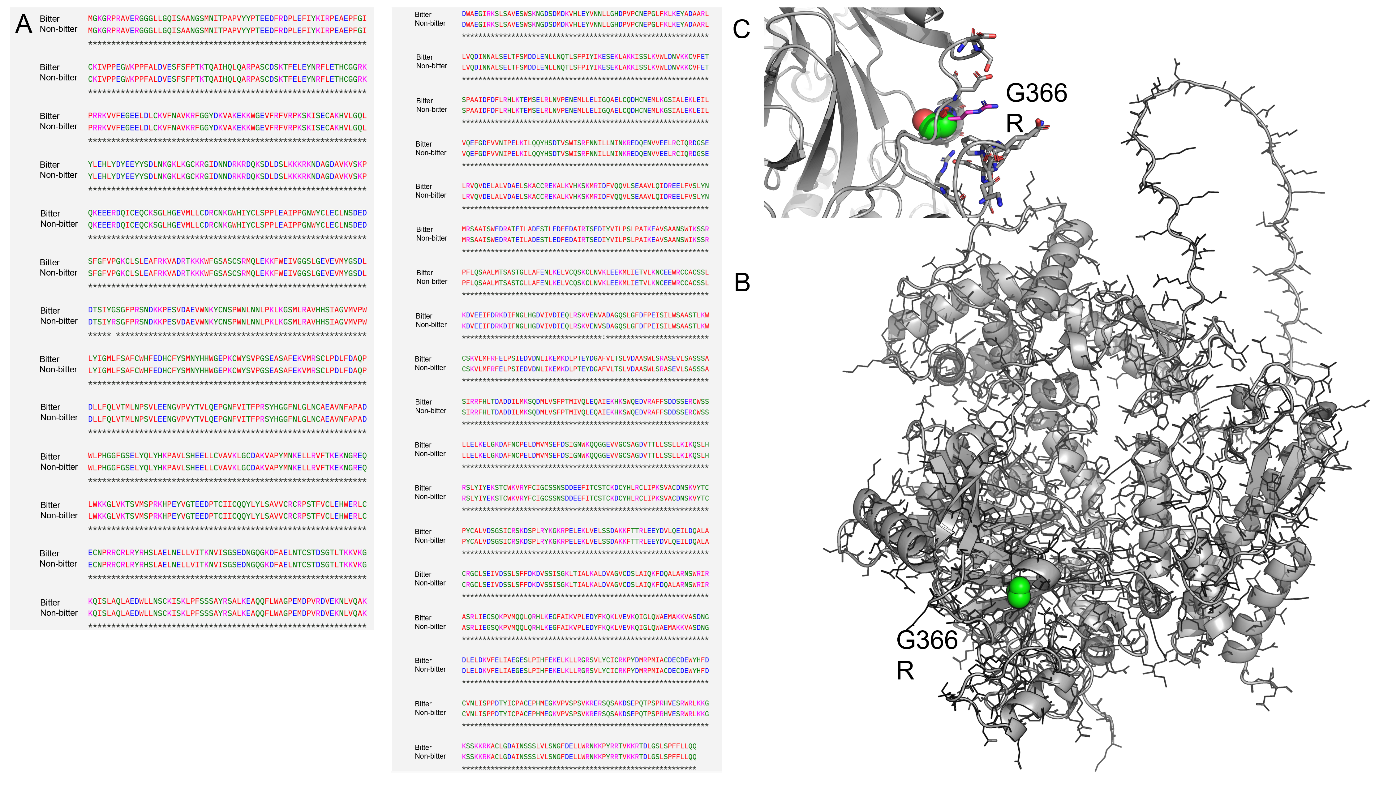


**Figure S8: AUR62017213.** **A**, Sequence alignment. **B**, AlphaFold model of the bitter form. Substituted residues are shown as coloured sphere models. **C**, Zoom into the region of the variant.


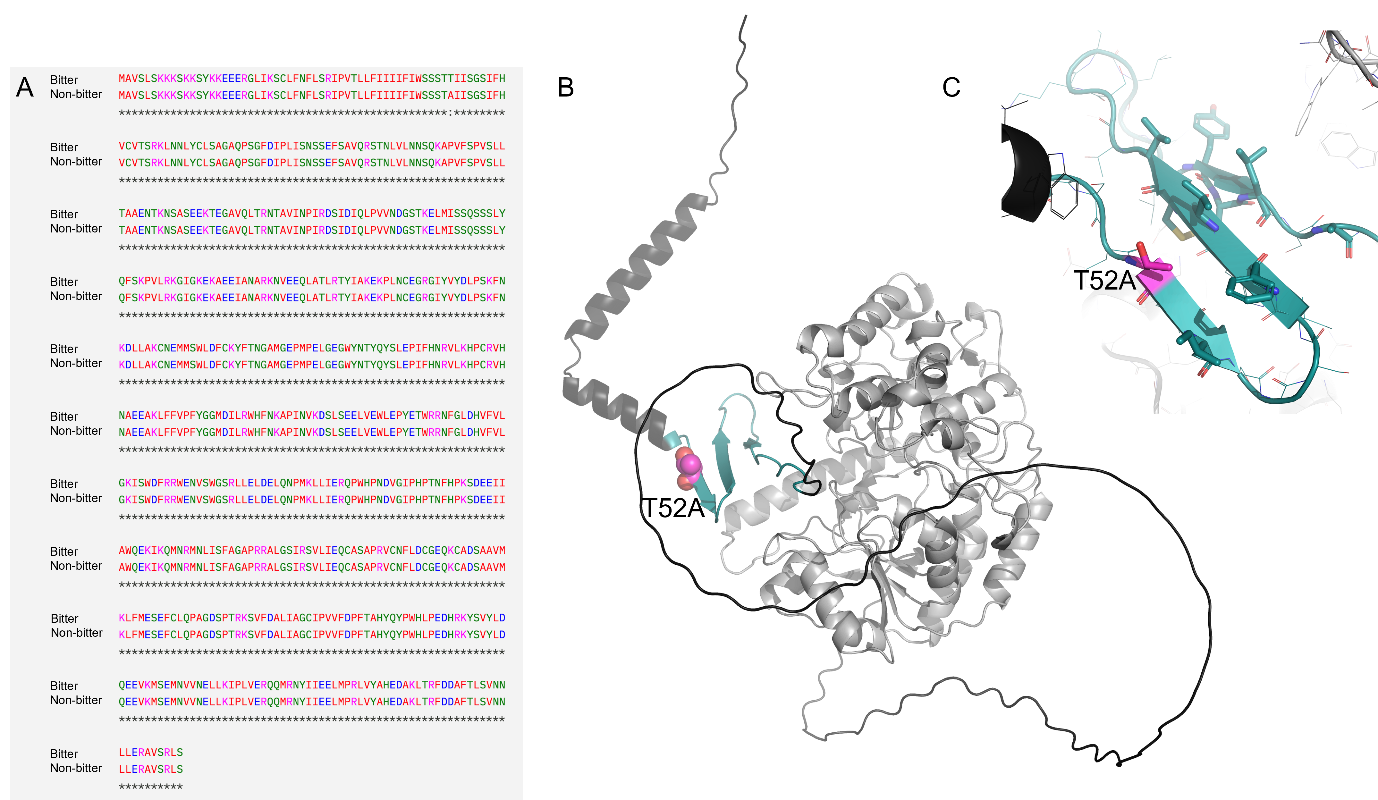


**Figure S9: AUR62017228.** **A**, Sequence alignment. **B**, AlphaFold model of the bitter form. Substituted residues are shown as coloured sphere models. **C**, Zoom into the region of the variant.


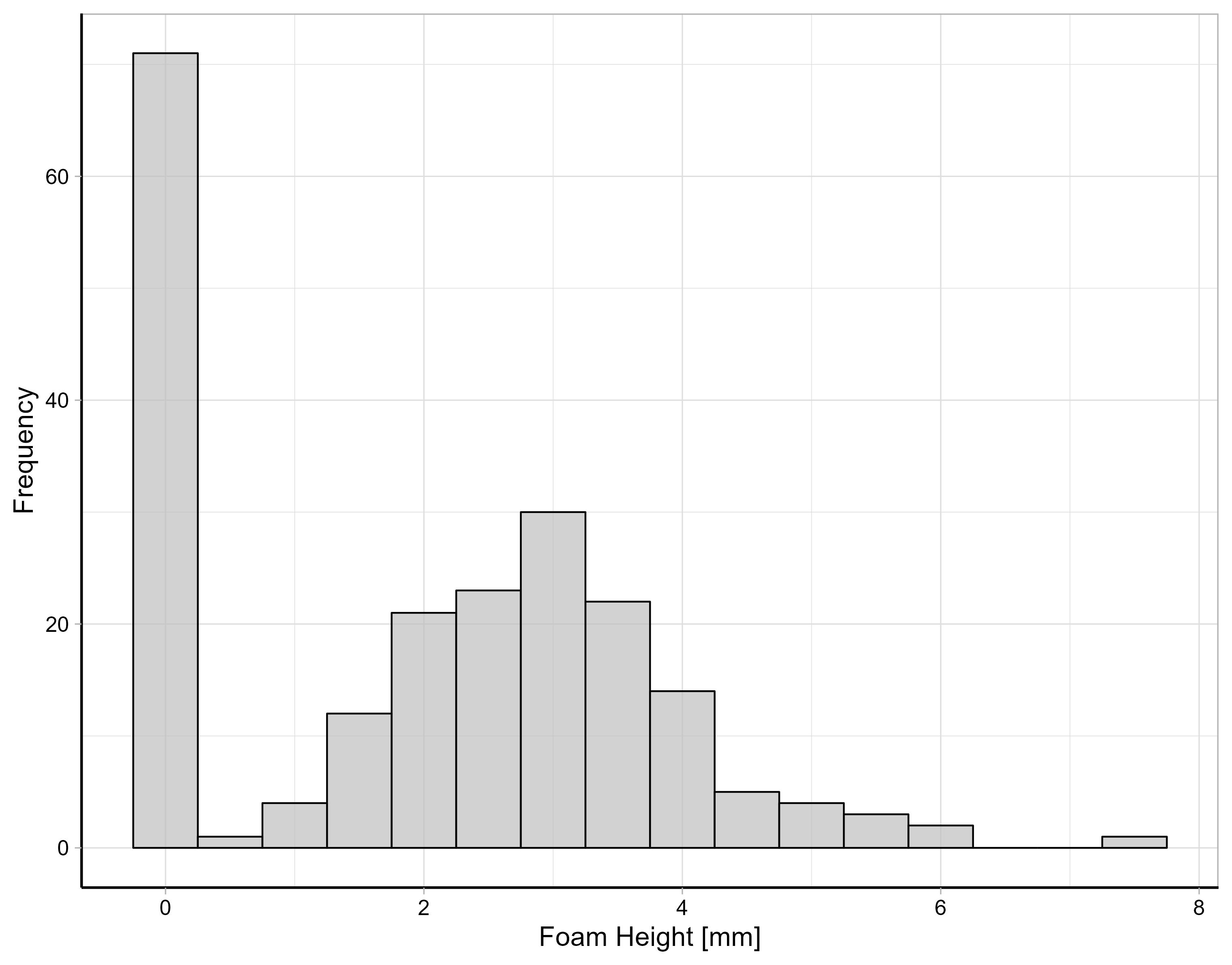


**Figure S10: Distribution of foam height.** Histogram of foam height measurements by afrosimetric method of *C. quinoa* accessions (n= 213).


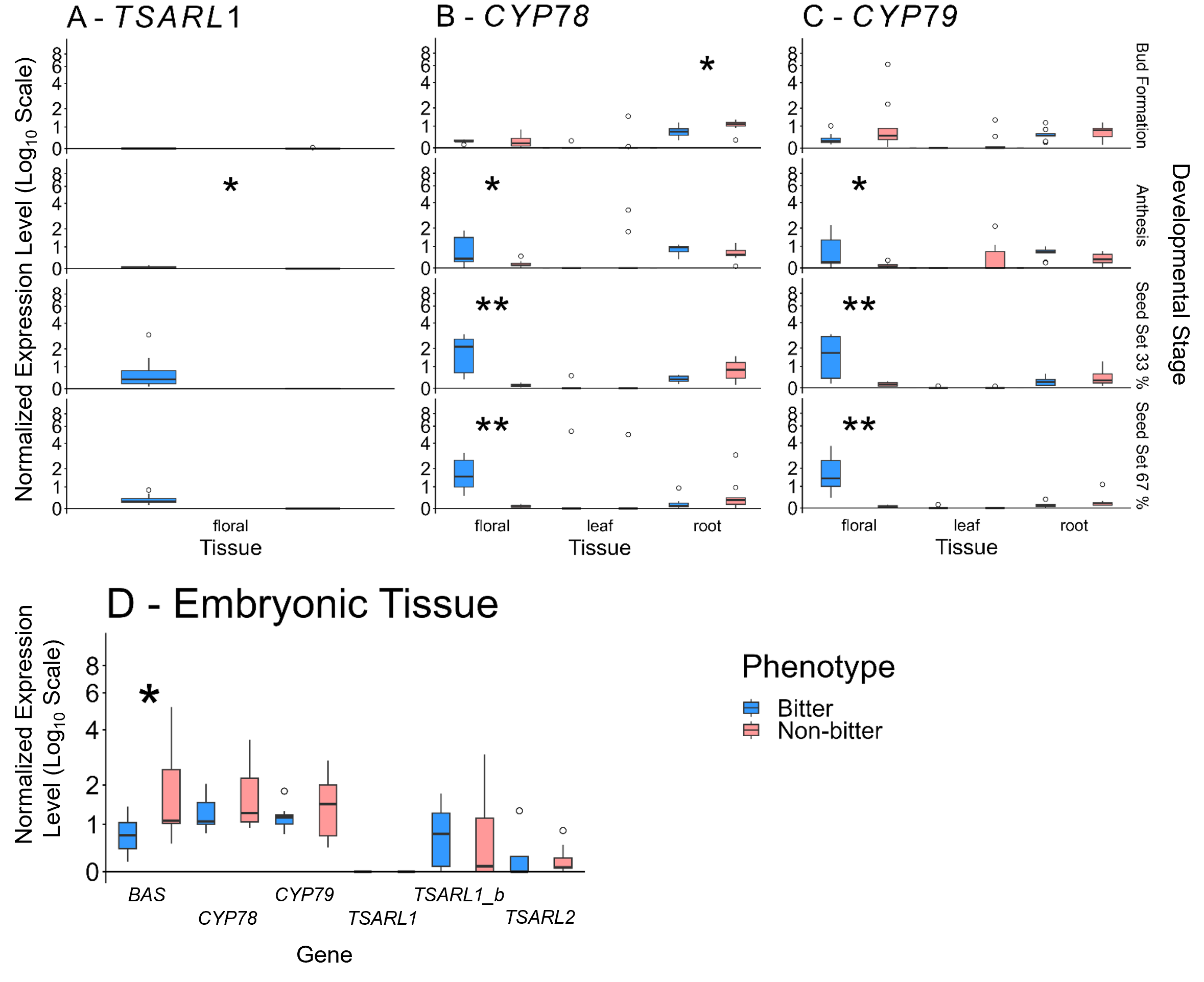


**Figure S11: Distribution of gene expression levels in bitter and non-bitter quinoa accessions of various proteins involved in saponin biosynthesis.**

Normalised expression levels are shown in blue (bitter quinoa accessions) and red (non-bitter quinoa accessions). Three different tissues (floral, leaf and root) and four different developmental stages (bud formation, anthesis, seed set 33 %, seed set 67 %) were analysed. **A** shows the gene expression of *TSARL1*, ‘bitter’ indicates that a primer pair was used for qPCR that is only supposed in binding to non-mutated *TSARL1* present in bitter quinoa accessions. **B** and **C** depict the expression of *CqCYP716A78* and *CqCYP716A79*, members of the CYP716 family involved in saponin biosynthesis. **D** displays the expression levels of these genes in the embryonic tissue. Per boxplot n is between 1 and 9. Significance levels of means are indicated by * (*p*-value ≤ 0.05), ** (*p*-value ≤ 0.01). An empty space indicates no significant difference between mean values.


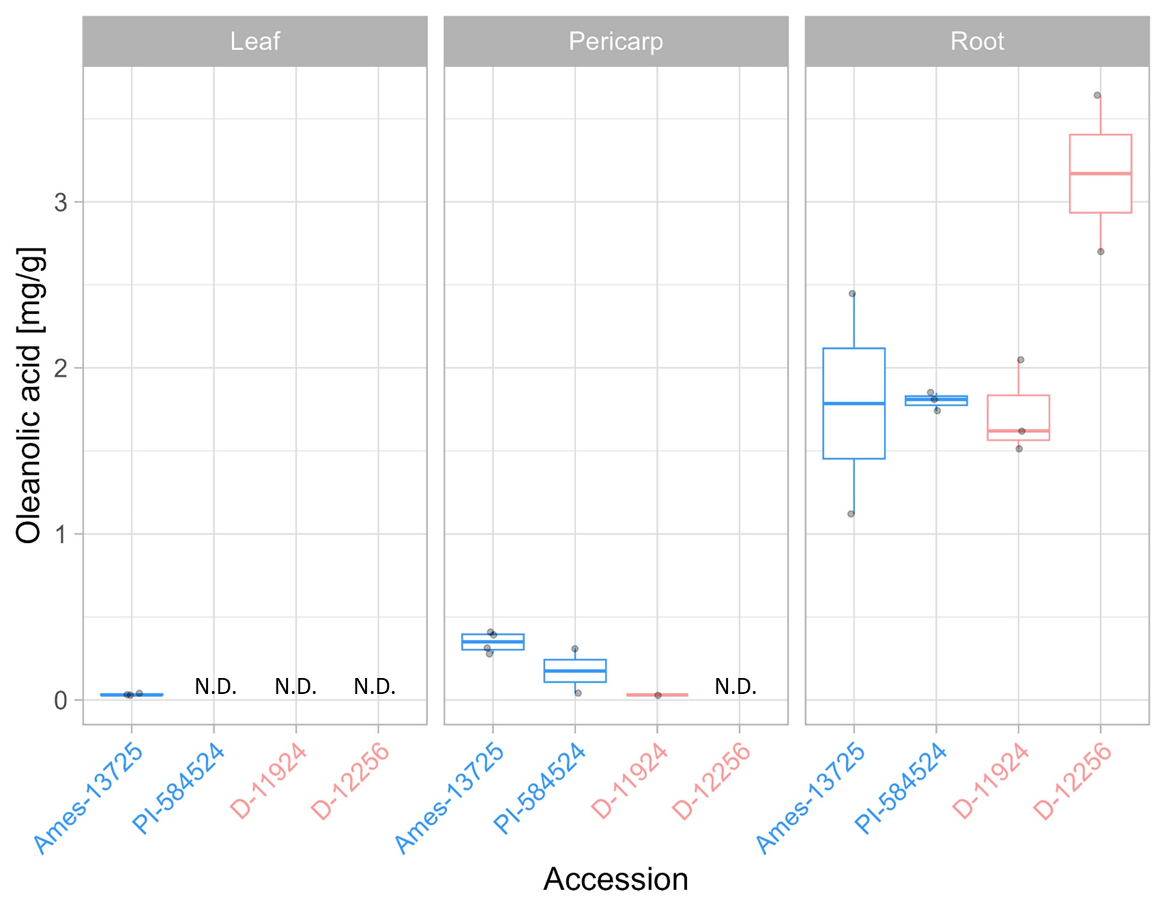


**Figure S12: Oleanolic acid content in different tissues of bitter and non-bitter quinoa accessions.** The oleanolic acid content in three different tissues (leaf, pericarp, and root) of two bitter quinoa accessions (blue; Ames-13725, PI-584524) and two non-bitter quinoa accessions (red; D-11924, D-12256) is displayed (n=2). N.D. - not detected.

 
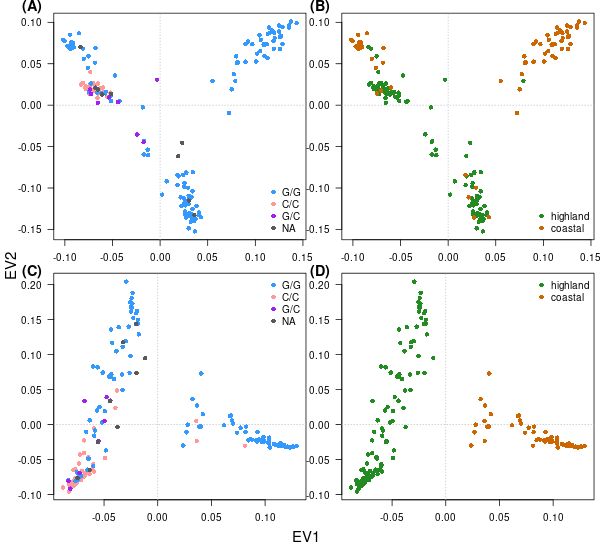


**Figure S13: Principal component analysis of the *TSARL1* gene region in comparison to the whole genome.** **A, B** Eigenvalues 1 and 2 plotted for the *TSARL1* gene region (EV1 = 21.92%, EV2 = 14.15%) and **C, D** for the whole genome (EV1 = 10.73%, EV2 = 4.34%), coloured according to the genotype at position 8,942,528 on Cq5B (**A**, **C**; G/G blue, C/C red, G/C purple, missing data grey) and the ecotype (**B**, **D**; highland in green, coastal in dark orange).

**Table S1: *C. quinoa* accessions** with information on origin, supplier, sequencing information, phenotype, genotype, foam data and GC-MS values. ‘b’ and ‘s’ indicate bitter and non-bitter (sweet) quinoa accessions respectively.

**Table S2: Scoring of emergence time and true leaf development.** Table contains data on investigated accessions, categorised by saponin phenotype and site of origin, along with recorded scores for emergence time and the appearance of first true leaves.

**Table S3: Sequences for genes with primer pairs used for qPCR.** ‘s/b’ and ‘b’ indicate that primer pairs were used to either bind to both bitter and non-bitter quinoa *TSARL1*, or only the non-mutated *TSARL1* present in bitter quinoa accessions.

**Table S4: qPCR samples and quinoa accession information.** Table shows information about the samples used for qPCR regarding gene, tissue, phenotype, developmental stage, replicate, Cq-value, Q (relative quantity of gene expression), NF (normalization factor), and NEL (normalized expression level). Furthermore, quinoa accessions of which samples were taken are described regarding accession name, phenotype, origin, accession number, and supplier.
